# Prognostic Significance of EBV Latent Membrane Protein 1 Expression in Lymphomas: Evidence from 15 Studies

**DOI:** 10.1371/journal.pone.0060313

**Published:** 2013-04-17

**Authors:** Yuan Mao, Mei Ping Lu, Hong Lin, Da Wei Zhang, Ying Liu, Qing Dong Li, Zhi Gang Lv, Jia Ren Xu, Ren Jie Chen, Jin Zhu

**Affiliations:** 1 Department of Otolaryngology-Head and Neck Surgery, Jiangsu Province Official Hospital, Nanjing, China; 2 Huadong Medical Institute of Biotechnology, Nanjing, China; 3 Department of Otolaryngology-Head and Neck Surgery, The First Affiliated Hospital of Nanjing Medical University, Nanjing, China; 4 Jiangsu Provincial Blood Center, Nanjing, China; 5 Department of Otolaryngology-Head and Neck Surgery, The Second Affiliated Hospital of Nanjing Medical University, Nanjing, China; 6 Department of Clinical Laboratory, Jiangsu Province Official Hospital, Nanjing, China; 7 Department of Hematology and Oncology, Jiangsu Province Official Hospital, Nanjing, China; 8 The Key Laboratory of Cancer Biomarkers, Prevention & Treatment Cancer Center and The Key Laboratory of Antibody Technique of Ministry of Health, Nanjing Medical University, Nanjing, China; The University of North Carolina at Chapel Hill, United States of America

## Abstract

**Background:**

Epstein-Barr virus (EBV) infection has been associated with lymphoma development. EBV latent membrane protein 1 (LMP1) is essential for EBV-mediated transformation and progression of different human cells, including lymphocytes. This meta-analysis investigated LMP1 expression with prognosis of patients with lymphoma.

**Methods:**

The electronic databases of PubMed, Embase, and Chinese Biomedicine Databases were searched. There were 15 published studies available for a random effects model analysis. Quality assessment was performed using the Newcastle-Ottawa Quality Assessment Scale for cohort studies. A funnel plot was used to investigate publication bias, and sources of heterogeneity were identified by meta-regression analysis. The combined hazard ratios (HR) and their corresponding 95% confidence intervals of LMP1 expression were calculated by comparison to the overall survival.

**Results:**

Overall, there was no statistical significance found between LMP1 expression and survival of lymphoma patients (HR 1.25 [95% CI, 0.92–1.68]). In subgroup analyses, LMP1 expression was associated with survival in patients with non-Hodgkin lymphoma (NHL) (HR  = 1.84, 95% CI: 1.02–3.34), but not with survival of patients with Hodgkin disease (HD) (HR  =  1.03, 95% CI: 0.74–1.44). In addition, significant heterogeneity was present and the meta-regression revealed that the outcome of analysis was mainly influenced by the cutoff value.

**Conclusions:**

This meta-analysis demonstrated that LMP1 expression appears to be an unfavorable prognostic factor for overall survival of NHL patients. The data suggested that EBV infection and LMP1 expression may be an important factor for NHL development or progression.

## Introduction

Lymphoma, the most common form of hematological malignancy in the world, originates from lymphocytes [1]. Lymphoma can be classified into Hodgkin disease (HD) and non-Hodgkin lymphoma (NHL), while NHL can be further divided into natural killer cell and T cell lymphoma (NK/TCL), and B cell lymphoma [1]. Lymphoma is one of the top ten most frequently diagnosed cancers and cancer deaths in China. The incidence and mortality of lymphoma in the Chinese population is approximately 6.06/100,000 and 3.64/100,000, respectively [2]. Among different risk factors for developing lymphoma, Epstein-Barr virus (EBV) latent infection has been shown to plays an important causitive role, and EBV has been implicated in development of a wide range of lymphoproliferative disorders [3], [4]. EBV infection has been reported to be associated with Hodgkin's lymphoma, Burkitt's lymphoma, and central nervous system lymphoma [1]–[4]. However, the mechanism of how EBV infection causes lymphomas remains to be elucidated.

Latent membrane protein 1 (LMP1) is an integral transmembrane protein that is encoded by EBV and contains three domains: an N-terminal cytoplasmic tail, six transmembrane-spanning loops, and a C-terminal cytoplasmic region [5]. Functionally, LMP1 is essential for EBV-mediated growth transformation of infected cells, and the C-terminal region of LMP1 protein can trigger a variety of signaling pathways in cells such as NF-κB and JAK/STAT to regulate the cell proliferation, immortalization, and invasion of lymphoma cells [6], [7]. Therefore, LMP1 expression has been suggested to have oncogenic effect in the development and progression of EBV-related lymphomas [8], [9]. Thus, clinically, detection of LMP1 expression is attracting considerable attention as a prognostic predictor and a novel target for anti-lymphoma therapy [10]. However, to date, there has been no systematic study to estimate the prognostic impact of LMP1 expression on clinical outcome of lymphoma. Further, studies are lacking on approaches to target LMP1 protein to effectively prevent EBV infection or eliminate the effects of EBV on lymphocytes.

To explore the association between LMP1 expression and clinical outcome in lymphoma patients, we performed a meta-analysis. We collected 15 published studies to objectively evaluate the prognostic significance of LMP1 expression in patients with lymphoma. Furthermore, the quality and publication bias of this study, and the extent and sources of heterogeneity in the published literature were also evaluated and analyzed.

## Materials and Methods

### Literature search and study selection

The electronic databases of PubMed, Embase, and Chinese Biomedicine Databases were searched for published studies that investigated the prognostic significance of LMP1 in lymphoma to be able to include in this meta-analysis (last search was performed in April, 2012). Research work was examined without language limits, and were identified by using the following keywords: “lymphoma,” “lymphoproliferative disorders,” “EBV,” “LMP1,” “biomarker,” “survival,” “prognostic factor”, and “prognosis” separately and in combination. The references of all publications and reviews were then reviewed and re-searched to prevent missing any relevant publications.

### Study inclusion/exclusion criteria

Studies eligible for inclusions in this meta-analysis were based on the following criteria: 1) Evaluated expression or amplification of LMP1 in lymphoma tissues by using immunohistochemistry (IHC) or quantitative real-time polymerase chain reaction (q-PCR); 2) Association of LMP1 expression or amplification with overall survival of lymphoma patients; and 3) Statistical analysis of hazard ratios (HRs) for overall survival based on LMP1 expression or amplification, which was either reported directly or was able to be retrieved from the data provided in the studies. However, if the studies on the same patient populations were reported in different publications, only the latest or the most completed publication was included in this meta-analysis. Two researchers independently assessed eligibility of every study and resolved any disagreements by a consensus reviewer.

### Quality assessment of primary publications

Quality assessment was first performed in each of the included publications by two independent reviewers using the Newcastle-Ottawa Quality Assessment Scale for cohort studies (Table 1) [11]. This scale is an eight-item instrument to allow assessment of patient population and selection, study comparability, follow-up, and outcome of interest. Interpretation of this scale was performed by awarding points, or ‘stars’, for high-quality elements. Stars were then added up and used to compare study quality in a quantitative manner.

**Table 1:** Newcastle-Ottawa quality assessment scale.

Contents	Items
Selection	(1) Representativeness of the exposed cohort
	a) truly representative of the average lymphoma patients (describe) in the community*
	b) somewhat representative of the average lymphoma patients in the community*
	c) selected group of users (e.g., nurses, volunteers)
	d) no description of the derivation of the cohort
	(2) Selection of the non exposed cohort
	a) drawn from the same community as the exposed cohort*
	b) drawn from a different source
	c) no description of the derivation of the non exposed cohort
	(3) Ascertainment of exposure (*Proof of lymphoma and LMP1 measurement*)
	a) secure record (e.g., surgical records)*
	b) structured interview*
	c) written self report
	d) no description
	(4) Demonstration that outcome of interest was not present at start of study
	a) yes*
	b) no
Comparability	(1) Comparability of cohorts on the basis of the design or analysis
	a) study controls for metastasis or recurrence*
	b) study controls for any additional factor (*Age, stage, type, etc*)*
Outcome	(1) Assessment of outcome (*Death or recurrence*)
	a) independent blind assessment*
	b) record linkage*
	c) self report
	d) no description
	(2) Was follow-up long enough for outcomes to occur (*Death or recurrence*)
	a) yes (3 year )*
	b) no
	(3) Adequacy of follow up of cohorts
	a) complete follow up – all subjects accounted for*
	b) subjects lost to follow up unlikely to introduce bias – small number lost – (25%) follow up, or description provided of those lost*
	c) follow up rate (<75%) and no description of those lost
	d) no statement

A study can be awarded a maximum of one star (*) for each numbered item within the Selection and Outcome categories. A maximum of two stars can be given for Comparability. Underlined and quoted phrases are provided in the scale to allow for adjustment to particular studies. Text in italic indicated our interpretation of the question relevant to this study.

### Definition of outcomes and comparisons

The primary data consisted of overall survival in all studied populations, and further subgroup comparisons that were carried out by ethnicity, histological types, cutoff value, HR estimate, the primary antibodies used, literature written language and statistical methods. The effective value on overall survival was determined by the combination of the log hazard ratio (HR) and associated standard error. If the HR data and 95% confidence intervals (CI) were not directly reported in the original studies, such data were then extracted from the Kaplan-Meier survival curve and estimation of the HR was performed using the method of Tierney et al [12], or data were calculated from other available information in the original studies by following the process described by Parmar et al [13].

### Statistical analysis

Statistical heterogeneity assessment was performed by using a χ2-based test and evaluation of the inconsistency index (I^2^) [14]. The I^2^ statistic is defined as the percentage of variability due to heterogeneity rather than the chance, with values>50% representing the possibility for substantial heterogeneity; in such a case, the random-effects model was used [15]. Otherwise, a fixed effects model was used (I^2^≤50%) [16]. The combination of the estimated risk was obtained by calculating a weighted average of the log (HR) estimates. A combined HR>1 implied a worse survival for the group with LMP1 overexpression. The unfavorable impact of LMP1 on survival of lymphoma patients was considered as statistically significant if the 95% CI for the combined HR did not overlap 1. The significance of the pooled HR was determined by the Z test, and a *p*-value of less than 0.05 was considered as statistically significant. Assessment of publication bias was performed for each of the pooled studies using the Egger’s and the Begg’s test [17]. Quantitative evaluation of sources of heterogeneity was performed by meta-regression analysis [18]. For assessing the stability of the outcomes, sensitivity analysis was performed by deleting each individual study in the meta-analysis to detect the influence of a single data set on the pooled HR [19]. Statistical analyses were carried out by using STATA Version 12.0 (Stata Corporation, College Station, TX).

## Results

### Selection and characteristics of the published studies

**Figure 1:**
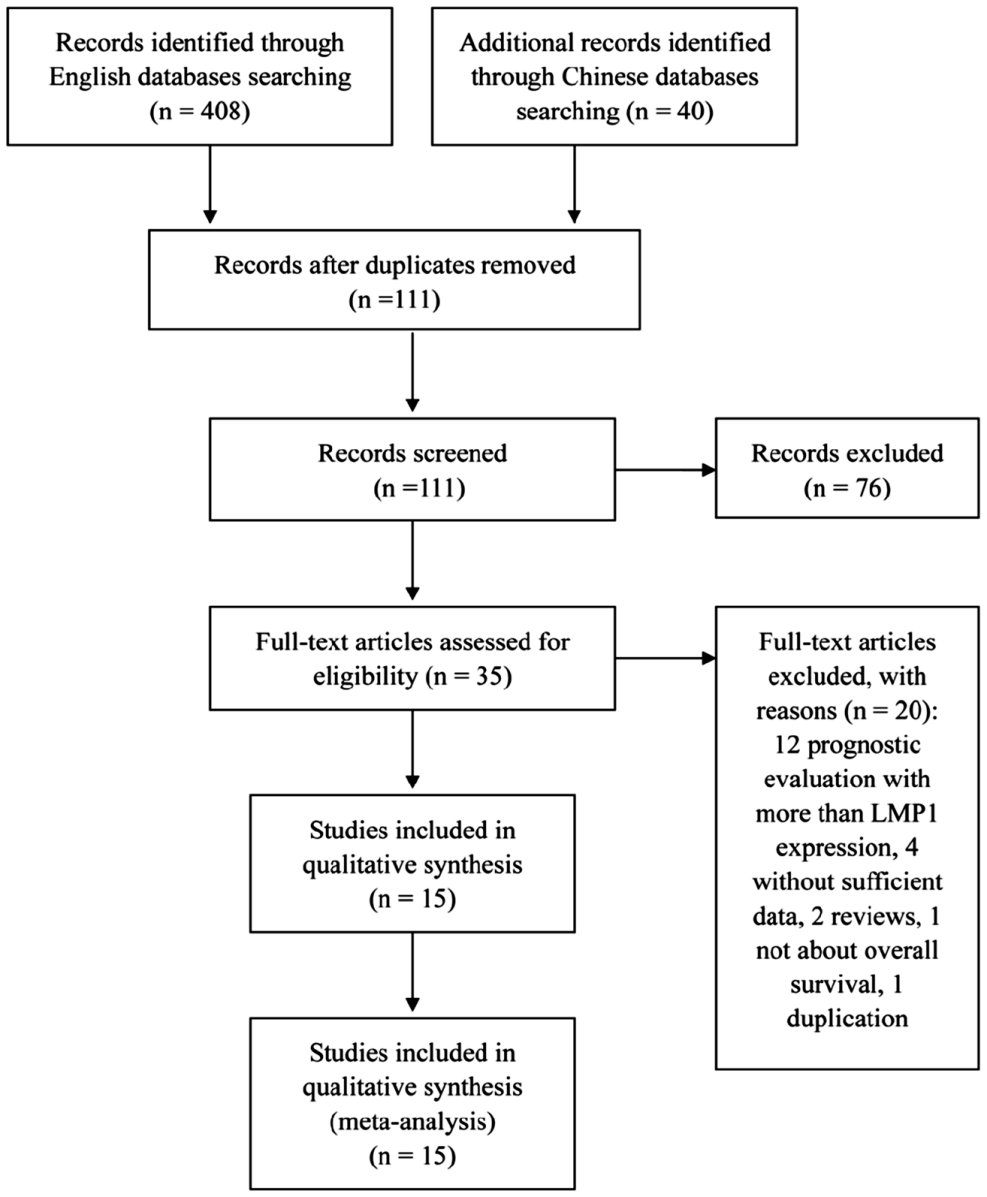
PRISMA flow diagram for selection of studies in the meta-analysis.

Through our initial literature search, we identified 111 studies that investigated the association of LMP1 and lymphoma. After screening the publication titles or abstract by two investigators, 76 studies were excluded because these publications were irrelevant to the current analysis, review articles, *in vitro* studies, or duplicate reports. The remaining 35 potentially eligible studies [20]–[54] were subjected to full-text evaluations. We found that 12 studies not only evaluated prognosis value of LMP1 expression, but also other parameters, such as the association between the expression of EBV-encoded Epstein-Barr virus early RNAs (EBERs) and the prognosis of lymphoma; thus, data on LMP1 expression and survival were not able to be specifically retrieved [35]–[46]; 4 studies were lacking relevant and convincing survival data [47]–[50]; 2 studies were review papers without actual data [51], [52]; one study was lack of data on overall survival [53], and another study was duplicate reported [54]. Thus, we obtained 15 studies with 2288 patients that met our inclusion criteria for this meta-analysis [20]–[34]. The study design and procedures were shown in Figure 1 and the main outcome data of these 15 studies were listed in Table 2. The points of study quality assessed by Newcastle-Ottawa quality assessment scale ranged from 3 to 7 (with a mean of 4.93), with higher value indicating better methodology.

**Table 2:** Summary of the eligible studies in this meta-analysis.

Study	Ethnicity	Histology	Stage	Cutoff Value	Written Language	No. Patients	Positive	Method	Antibody	HR Estimate	HR	95% CI	Study Quality (points)	Prognostic Significance
Kanemitsu 2012 [20]	Japan	NHL(NK/TCL)	I–IV	NA	English	30	73.3%	IHC	CS1-4 (Dako)	OS	0.19	0.04–0.95	6	Negative
Paydas 2008 [21]	Turkey	NHL	I–IV	1	English	148	16.9%	IHC	CS1-4 (Novacastra)	Log rank+P	4.39	2.38–8.10	5	Positive
Cao 2008 [22]	China	NHL(NK/TCL)	I–IV	10	English	58	81%	IHC	CS1-4 (Dako)	Log rank+P	2.06	1.01–4.22	7	Positive
Ishii (2007) [23]	Japan	NHL(NK/TCL)	I,II,IV	40	English	20	35%	qPCR	NA	Log rank+P	6.41	1.86–22.14	5	Positive
Cao 2003 [24]	China	NHL	I–IV	5	Chinese	65	70.8%	IHC	NA	Log rank+P	1.99	1.01–3.55	3	Positive
Keresztes 2006 [25]	Hungary	HD	I–IV	NA	English	109	43.1%	IHC	Mice monoclonal antibody(Dako)	Log rank+P	1.34	0.89–2.00	5	Indeterminate
Claviez 2005 [26]	Germany-Austria	HD	I–IV	NA	English	842	31.2%	IHC	CS1-4(Dako)	Log rank+P	1.18	1.03–1.35	5	Positive
Quijano 2004 [27]	Colombia	HD	I–IV	NA	Spanish	57	56.1%	IHC	CS1-4(Dako)	Log rank+P	0.69	0.40–1.19	4	Indeterminate
Krugmann 2003 [28]	Austria	HD	I–IV	NA	English	119	26.1%	IHC	CS1-4(Dako)	OS	1.72	0.71–4.19	5	Indeterminate
Herling 2003 [29]	Sweden	HD	I–IV	NA	English	303	20.1%	IHC	CS1-4 (Dako)	OS	1.34	0.42–4.30	6	Indeterminate
Stark 2002 [30]	England	HD	I–IV	NA	English	70	34.3%	IHC	E29 (Dako)	OS	3.2	1.28–8.02	4	Positive
Glavina-Durdov 2001 [31]	Croatia	HD	I–IV	NA	English	100	26%	IHC	CS1-4 (Dako)	OS	0.58	0.24–1.41	5	Indeterminate
Montalban 2000 [32]	Spain	HD	I–IV	NA	English	110	53.6%	IHC	CS1-4 (Dako)	RP	0.35	0.13–0.90	5	Negative
Enblad 1999 [33]	Sweden	HD	I–IV	NA	English	117	27.4%	IHC	CS1-4 (Dako)	OS	2.36	0.81–6.82	5	Indeterminate
Morente 1997 [34]	Spain	HD	I–IV	NA	English	140	51.4%	IHC	CS1-4 (Dako)	RP	0.39	0.17–0.92	4	Negative

NHL = non-Hodgkin lymphoma; HD = Hodgkin lymphoma or Hodgkin disease; NK/TCL = natural killer cell and T cell lymphoma; DLBCL = diffuse large B cell lymphoma; IHC = immunohistochemistry; qPCR =  quantitative real-time polymerase chain reaction; OS = overall survival curve; RP = HR reported directly; HR =  hazard ratios; NA = not available. Study quality is listed using the results of the Newcastle -Ottawa questionnaire (Table 1). Summary results were either positive (95% CI above 1.0) or negative (95% CI below 1.0) or indeterminate (95% CI crossing 1.0).

In brief, we found that of these 15 publications, four studies were conducted in Asia, and 11 studies were conducted in either Europe or South America (non-Asian). 13 studies were written in English, one in Chinese and one in Spanish. In terms of histology of lymphoma studied, five publications reported NHL (three were NK/TCL and two were mixed T and B cell lymphomas and rest of the 10 studies were HD. Furthermore, 14 studies investigated LMP1 expression in stage I-IV lymphomas using immunohistochemistry, where one study detected LMP1 expression using q-PCR in stage I-II and IV lymphomas. Of 14 studies with immunohistochemistry, 11 assessed LMP1 expression by using the monoclonal CS1-4 antibody as the primary antibody. However, only four studies provided the accurate cutoff value for positive cytoplasmic staining of LMP1 in lymphoma tissues. In addition, three studies reported LMP1 as a predictor of favorable prognosis, whereas six studies suggested LMP1 protein as the poor prognosis indicator, but the remaining six studies showed no significant impact of LMP1 expression on overall survival of lymphoma patients.

### Meta-analysis

**Figure 2:**
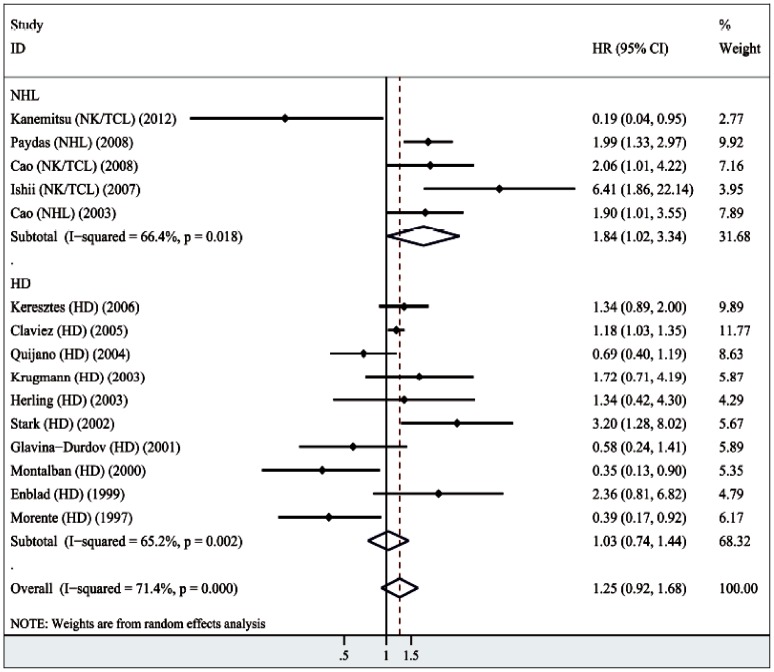
Forest plot of the hazard ratios (HR) and 95% confidence intervals (CI) of each study on association of LMP1 expression with lymphoma (random-effect model) and with histological types of lymphomas.

Using these 15 studies we conducted quantitative aggregation of the survival data, the main results of each meta-analysis are presented in Table 2. Since significant heterogeneity was detected in these studies, we performed the random-effects model analyses of these 2288 patients. The combined HR was 1.25 ([95% CI: 0.92–1.28]; I^2^ = 71.4%) of LMP1 expression in lymphoma (Figure 2). Furthermore, there was no statistical association of LMP1 expression with worse prognosis of all lymphoma patients. If grouped according to histology, the combined HR of NHL subtype was 1.84 (95%CI: 1.02–3.34), suggesting a significant association of LMP1 expression with worse NHL prognosis, whereas the combined HR of HD patients was 1.03 (95%CI: 0.74–1.44), indicating lack of an association of LMP1 expression with HD prognosis.

Moreover, we performed subgroup analyses according to ethnicity, cutoff value of LMP1 expression levels, HR estimation and written language. The data are shown in Table 3. Briefly, there was a statistically significant association of LMP1 expression with overall survival of lymphoma patients from four studies that used a specific cutoff value for LMP1 expression (HR, 2.14 [95% CI: 1.55–2.95], I^2^ = 8.7%). However, such an association disappeared in the rest of 11 studies that did not use a cutoff value (HR, 0.97 [95% CI: 0.69–1.37], I^2^ = 67.5%). Moreover, the combined HR between LMP1 expression and overall survival of patients was 0.37 (95% CI: 0.20–0.70, I^2^ = 0.0%), when the survival data were directly reported in the studies. In contrast, when the survival data calculated through log rank plus P method, the combined HR of LMP1 expression was 1.51 (95% CI: 1.10–2.07, I^2^ = 71.0%). There was no association between LMP1 expression and survival of patients, when the survival data were indirectly calculated from Kaplan-Meier based survival curves (HR 1.24 [95% CI: 0.60–2.53], I^2^ = 64.6%). There was no statistical significance found between Asian (HR 1.69 [95% CI: 0.66–4.33], I^2^ = 74.7%) and non-Asian (HR 1.13 [95% CI: 0.82–1.54], I^2^ = 69.3%), or between English written studies (HR 1.27 [95%CI: 0.91–1.78], I^2^  = 71.9%) and non-English written studies (HR 1.13 [95% CI: 0.42–3.05], I^2^ = 82.4%).

**Table 3:** The results of summarized HRs in overall and subgroup analyses of survival and the results of meta-regression.

	No. Studies	No. Patients	LMP1:HR (95% CI)	Heterogeneity Test			Meta regression Test		
				χ^2^	I^2^	P-value	P-value	Ratio of HR	95% CI
All Studies in Lymphoma	15	2288	1.25 (0.92–1.68)	48.91	71.4%	0.000	-	-	-
Ethnicity									
Asian	4	173	1.69 (0.66–4.33)	11.87	74.7%	0.008	0.391	1	1
Non-Asian	11	2115	1.13 (0.82–1.54)	32.62	69.3%	0.000	-	0.67	0.47–3.40
Histology									
NHL	5	321	1.84 (1.02–3.34)	11.89	66.4%	0.018	0.216	1	1
HD	10	1967	1.03 (0.74–1.44)	25.83	65.2%	0.002	-	0.56	0.53–2.07
Cutoff Value									
Report	4	291	2.14 (1.55–2.95)	3.28	8.7%	0.350	0.043	1	1
Non-Report	11	1997	0.97 (0.69–1.37)	30.73	67.5%	0.001	-	0.45	0.60–1.54
HR Estimate									
HR Reported Directly	2	250	0.37 (0.20–0.70)	0.03	0.0%	0.869	0.271	1	1
HR Calculated from Survival Curves	6	739	1.24 (0.60–2.53)	14.13	64.6%	0.015	-	3.35	0.05–0.30
HR Calculated by Log rank+P	7	1299	1.51 (1.10–2.07)	20.72	71.0%	0.002	-	4.08	0.01–0.04
Written Language									
English written	13	2166	1.27(0.91–1.78)	42.72	71.9%	0.000	0.876	1	1
Non English written	2	122	1.13(0.42–3.05)	5.69	82.4%	0.017	-	0.89	0.41–4.74

NHL = non-Hodgkin lymphoma; HD = Hodgkin lymphoma or Hodgkin disease; NK/TCL = natural killer cell and T cell lymphoma; IHC = immunohistochemistry; HR =  hazard ratios.

### Heterogeneity and sensitivity analyses

Substantial heterogeneity was exhibited in overall HR of LMP1 expression in these lymphoma studies (I^2^ = 71.4%). To explore the potential sources of heterogeneity, we gathered information on ethnicity, histology, cutoff value, HR estimate and written language with the meta-regression model (Table 3). The meta-regression data showed that the consequence of analysis was mainly influenced by cutoff value (P = 0.043). Furthermore, in the sensitivity analysis, no individual study influenced the pooled HR qualitatively, which indicates the stable outcomes of this meta-analysis.

### Publication bias

**Figure 3:**
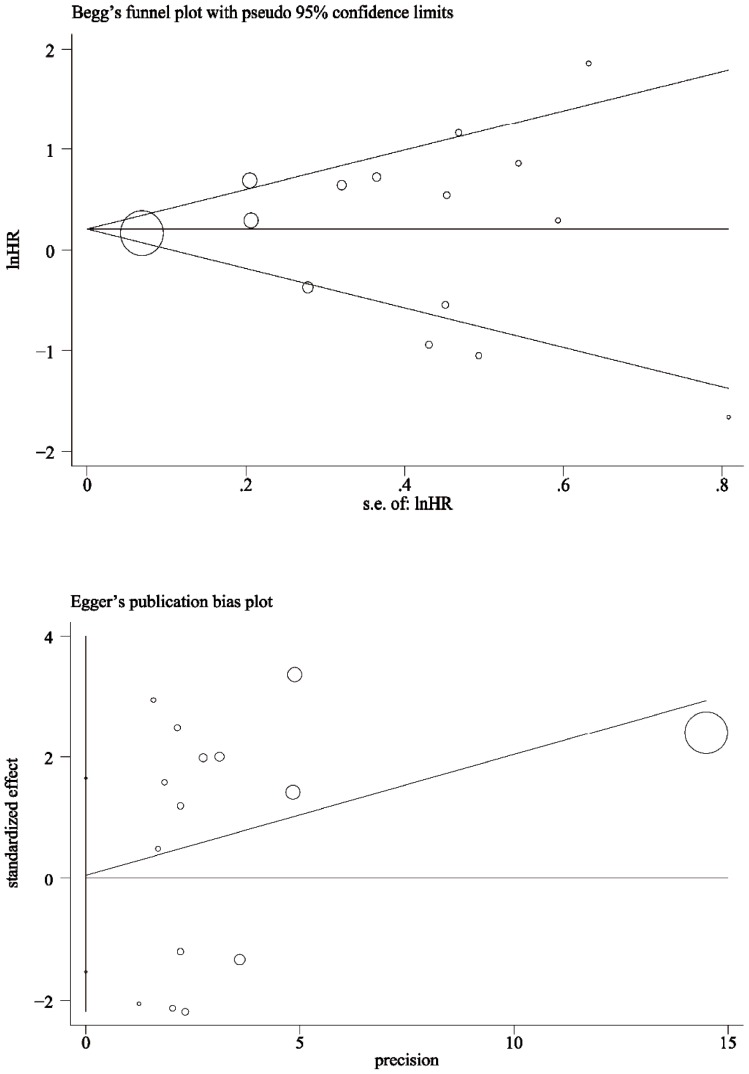
Funnel plots of Begg and Egger’s tests. Publication bias on overall estimation was evaluated. Studies are symmetrical distributed approximately above and below the horizontal line, and suggest the absence of publication bias in this meta-analysis.

The Egger’s test and Begg’s funnel plot were applied to evaluate publication bias in this meta-analysis. With all 15 included studies, no funnel plot asymmetry was found (p = 0.940 using the Egger’s test; Figure 3), indicating there was no evidence of publication bias detected in this study.

## Discussion

Meta-analysis is a method that contrasts and combines data from various individual studies to identify useful and insightful information on a particular topic. Advantages of meta-analysis include the ability to generalize data from individual studies with a greater number of studied subjects, higher statistical power, and being able to control for between-study variations and to show whether any publication bias exists. However, meta-analysis does have certain disadvantages. These disadvantages include the fact that the study is based on published data, which are not controlled by this analysis. Using this analysis, we are able to make much clearer conclusion for the association of LMP1 expression in lymphoma patients. The results of this meta-analysis containing 15 studies demonstrated that LMP1 expression was associated with over all survival of NHL patients, but not with HD patients. The data may indicate that EBV latent infection may promote NHL development or progression. Clinically, it is of a great interest to identify prognostic markers for patients with lymphomas to assist in clinical decision-making for therapy and prediction of outcomes. Indeed, the association of EBV infection with lymphoma has been studied for a long time and the importance of EBV infection has been shown to be limited to latent phase genes, and that LMP1 is one of the most significant oncogenic proteins that is linked to the etiology of lymphoma [7]. However, the prognostic value of LMP1 expression in lymphoma is still inconclusive and even controversial. Thus, our current data showed expression of LMP1 protein was just associated with overall survival of NHL patients. Indeed, our unpublished *ex vivo* data showed high expression of LMP1 protein in surgically resected NK/TCL lymphoma tissues using immunohistochemistry. In addition, we have tried to generate different polyclonal or monoclonal anti-LMP1 antibodies to assess their antitumor effects in nasopharyngeal carcinoma [55], [56].

There was substantial heterogeneity among the studies in this meta-analysis. For example, the characteristics of LMP1 in predicting prognosis of lymphoma from these 15 studies were contradictory, i.e., three studies reported LMP1 as a predictor of favorable prognosis, whereas six studies suggested LMP1 protein as the poor prognosis indicator, but the remaining six studies showed no significant impact of LMP1 expression on overall survival of lymphoma patients. Therefore, in this meta-analysis, we used a random effect model to assess the true value of LMP1 protein expression as a prognostic predicator. We found that the potential cause of heterogeneity could be the different cutoff values of LMP1 expression levels used in different studies. For example, the method used to detect LMP1 expression in lymphoma was immunohistochemistry in 14 of 15 studies. It is usually needed to make a cutoff value to evaluate immunoreactivity of any protein expression in tissue specimens. Thus, the subjective cut-off values in each study affected the overall data on study of LMP1 expression in lymphomas. Similarly, one study using q-PCR to detect expression of LMP1 DNA also applied a cutoff value. To date, there is unfortunately no standardized scoring system for detection and evaluation of gene expression using immunohistochemistry or q-PCR. Instead, this analysis often comes with arbitrary scoring system to set cutoff values for determining positivity [57], [58]. In these 15 studies, only four studies presented unambiguous cutoff values, while the rest did not. Thus, substantial heterogeneity occurred. In addition, we also tried to perform a fixed effect model using the 15 studies to assess the value of LMP1 protein expression in lymphoma. The results were contradictory in which statistical significance was not only witnessed between LMP1 expression in survival of lymphoma patients, but also in patients with non-Hodgkin lymphoma (NHL) and Hodgkin disease (data not shown). Because of the solid presence of heterogeneity, the fixed effect model for this meta-analysis was not suitable.

Another potential and trivial source of bias is related to the method of HR and 95% CIs extrapolation, because not all 15 studies showed HR and 95% CIs values directly and accurately. Thus, if the necessary statistical methods were not reported in the studies, we acquired data by using the survival curves [12] or we calculated the data from processing the available data in the studies [13]. This approach results in slightly different data; for example, using pooled data from reporting HRs directly, we obtained an HR of 0.37 [95% CI: 0.20–0.70], while using pooled data from reporting survival curves, we had an HR of 1.24 [95% CI: 0.60–2.53]. In contrast using pooled data from calculating with accessible data, we had an HR of 1.51 [95% CI: 1.10–2.07].

In addition, a publication bias remains a major concern in evaluating and validating data from these studies. Egger’s test was applied in this meta-analysis, and we found no evidence showing that publication bias may be significantly influencing our results. Furthermore, some limitations did occur in the current meta-analysis, e.g., i) The number of original studies included for analysis was small; ii) The histological types of lymphoma are complicated and diversified, which were far more than two types that we categorized in this study; iii) We did not collect unpublished abstracts for this analysis. In addition, there were four studies that didn’t provide sufficient data for us to calculate HRs and their corresponding CIs. This might influence the overall results and should be taken into account; and iv) An important issue that we need to face is the type of adjuvant therapy each patient received [59], [60], which may also affects the prognosis of the patients; however, the majority of published studies did not disclose the information on patient treatment. All of these limitations could contribute to additional inconsistencies and creation of potential selection bias. Thus, our current data need to be substantiated by adequate prospective studies.

In conclusion, this meta-analysis did not reach an overall conclusion that LMP1 expression is associated with lymphoma prognosis; however LMP1 expression may have a detrimental effect on survival of NHL patients. This piece of data could help us further investigate the role of EBV infection in NHL patients, such as design of a molecular targeted therapy for NHL. Lastly, our current data needs to be verified by using a large well-designed prospective study.
